# To what extent are vulnerability issues included and addressed in Kenya's health and immunization policy documents? A systematic review of documents

**DOI:** 10.3310/nihropenres.14010.1

**Published:** 2025-08-01

**Authors:** Esther Awuor Owino, David Mafigiri, Dorcas Kamuya, Caroline Jones, Primus Chi

**Affiliations:** 1 Centre for Geographic Medicine Research (Coast), Kenya Medical Research Institute-Wellcome Trust Research Programme, Kilifi, P.O. Box 230-80108, Kenya; 2 Makerere University, Kampala, Central Region, P.O. Box 7062, Uganda; 3 Centre for Tropical Medicine and Global Health, Nuffield Department of Medicine, University of Oxford, Oxford, England, OX3 7LG, UK

**Keywords:** Vulnerability, vaccine vulnerability, immunization, vaccination, vulnerable groups, vulnerable populations, document review, policy review, Kenya

## Abstract

**Introduction:**

Globally, childhood immunization is one of the most important public health interventions contributing to a significant reduction in childhood mortality and morbidity. This achievement has been made possible by several concerted efforts at the international and national levels. However, challenges persist, including disparities in vaccine coverage, consequently increasing vaccine vulnerability. This review aimed to examine how vulnerability issues are framed and addressed in Kenya's health sector and immunization policy documents.

**Methods:**

The Preferred Reporting Items for Systematic reviews and Meta-Analyses (PRISMA) guidelines guided the review process. Policy documents were retrieved from online searches, searching through the reference list of retrieved documents and requesting relevant documents from stakeholders. To select documents, we screened the titles and executive summaries of documents guided by the exclusion and inclusion criteria. Data was extracted using a data extraction template prepared in Excel, capturing the general information about the documents and the specific information about vulnerability. The extracted data was then organized thematically to address the review objectives.

**Results:**

Twenty-one documents were included for final review. Of these, four were immunization programme documents, 15 were documents that cut across the entire health sector and two were legislative documents. Across the documents, different vulnerable groups were outlined. We developed four typologies of vulnerability from the groups listed in the documents, namely: vulnerability as socio-economic condition; vulnerability as biological and health condition; and vulnerability as a physical location. Some of the strategies proposed in the documents to address vulnerability issues included, adopting a rights-based approach to service provision, removing financial barriers and conducting immunization outreach activities.

**Conclusion:**

Future policy development should recognize the overlapping and intersecting nature of vulnerability factors and develop comprehensive and flexible approaches to address various forms of vulnerability.

## Plain Language Summary

Childhood immunization plays an important role in preventing childhood diseases and deaths. This has been possible because of many measures that have been put in place by different countries. However, several countries are still experiencing challenges related to childhood vaccine access, hence putting children who miss out on vaccines vulnerable to not fully enjoying the benefits of vaccines. We reviewed health sector policy documents in Kenya to understand how vulnerability has been framed and addressed in the documents.

We conducted online searches to retrieve relevant policy documents. In addition, we requested documents from stakeholders and searched through reference lists of identified documents. We then selected relevant documents by screening the titles and executive summaries of the documents. Using a template prepared in Excel, we extracted information relevant to the study objectives from the documents.

A total of 21 documents were included for final analysis. In these documents, different vulnerable groups were outlined. However, only individual-specific factors were used to define the vulnerable without recognizing the potentially overlapping and interacting nature of these factors. We therefore suggest that for future policy development, the multiple and intersecting nature of vulnerability factors should be considered. In addition, comprehensive and flexible strategies would be more suitable to address the co-existence of multiple factors that can prevent an individual not accessing health services like immunization.

## Introduction

Childhood immunization remains one of the most crucial public health interventions for protecting children against life-threatening infectious diseases ^1, 2^. Over the years, immunization has significantly reduced morbidity and mortality among millions of children globally ^2^. The WHO estimates that about 3.5 to 5 million deaths from diseases like diphtheria, tetanus, pertussis, influenza and measles are averted annually because of immunization. These successes can be attributed to several concerted efforts internationally and by individual countries. Key among these was the establishment of the Expanded Programme on Immunization by WHO in 1974 and the Global Alliance for Vaccines and Immunization (GAVI) in 1999.

Despite the gains in routine childhood immunization, challenges persist. These include disparities in coverage between and within countries, attaining and sustaining set coverage targets such as the 90% national vaccination coverage, and the existence of many under-vaccinated and zero-dose children ^3– 5^. According to the United Nations Children’s Fund (UNICEF), about 20.5 million children globally were reported to be either unvaccinated or under-vaccinated, and 14.3 million were considered to have not received any of the Diphtheria, Pertussis, and Tetanus (DTP) doses (zero-dose children) as of 2022. Studies have shown that key factors influencing sub-optimal vaccine coverage include displacements and insecurity in conflict areas, inadequate investment in national immunization programs, shortages of vaccines, and disease outbreaks, among others ^6, 7^. In most low and middle-income countries (LMICs) social and structural factors such as poverty, education levels, and gender, coupled with political, economic and health system issues, contribute to sub-optimal vaccine coverage amongst the most disadvantaged groups ^8– 10^. Consequently, this contributes to inequity and increases vaccine vulnerability. Under immunized and unvaccinated children are not only vulnerable to poor health but also future vaccine-preventable disease outbreaks.

As a concept, vulnerability has been defined in different ways since it is rooted in several disciplines. The United Nations Office for Disaster Risk Reduction (UNDRR) defines vulnerability as “the conditions determined by physical, social, economic and environmental factors or processes which increase the susceptibility of an individual, a community, assets or systems to the impacts of hazards.” In the health and healthcare context, vulnerability has mostly been conceptualized as the potential risk of developing certain diseases, health conditions or suffering from environmental hazards ^11– 13^. This susceptibility to harm because of various forms of hazards can exacerbate healthcare disparities, including those related to immunization. Analysing the policy context within which national EPI programmes operate can help identify where gaps may exist in the conceptualization of vulnerability and in the strategies proposed for reducing various forms of vulnerability. This review aimed to examine how vulnerability issues are framed and addressed in health sector and immunization policy documents in Kenya.

## Methods

**Patient and Public Involvement:** This was a review of policy documents; therefore, patients and the public were not involved in the review process.

The Preferred Reporting Items for Systematic reviews and Meta-Analyses (PRISMA) guidelines ( Extended data file 1) were used to guide the review process and reporting of the review results. The methods presented here have also been published as a protocol ^14^. The published protocol for this review aimed at examining how equity and vulnerability are conceptualized in health policy documents for Kenya and Uganda. However, the final review only focused on how vulnerability is addressed in the Kenyan health policy documents. This change was made to align the review with a broader primary study, which aims to examine vulnerabilities influencing access and uptake of childhood immunization in Kenya.

### i. Eligibility criteria

In this review, a policy document was defined as an action plan, a guideline or a planning document that contains the government’s intention concerning a particular health issue(s), including specific policies, guidelines, strategic plans and laws ^15^. The review included the following categories of documents: health sector policy documents (documents that cut across the entire health sector), immunization-specific documents (documents which focus on the immunization programme in Kenya), documents in English language and those developed between 2000 (to correspond with the establishment of GAVI, which has played a key role in promoting access to vaccines in most developing countries like Kenya) to 2023.

### ii. Sources of the policy documents

We conducted online searches of relevant websites and general Google searches to retrieve policy documents. The websites that were searched included the official Kenyan Ministry of Health website ( https://www.health.go.ke/) *, w*ebsites of international organizations working around childhood immunization namely; United nations children Fund (UNICEF) ( https://www.unicef.org/), Global Alliance for Vaccines and Immunization (GAVI) ( https://www.gavi.org/), World Health Organization (WHO) ( https://www.who.int/) and Save the Children Kenya ( https://kenya.savethechildren.net/). Since this review is part of a broader primary study within Kilifi County in Kenya, we also requested relevant policy documents from Kilifi County Department of Health. Reference lists of the documents were also searched to identify additional policy documents.

### iii. Search strategy

We used the following Boolean phrases for the online searches: vaccine OR vaccin* OR immunization OR immuniz* AND policy OR guideline OR plan AND Kenya, health AND policy OR guideline OR plan AND Kenya expanded programme on immunization OR EPI OR Kenya expanded programme on immunization or KEPI. In addition, we searched manually through the resources sections of the specific websites mentioned above.

### iv. Document selection process

EO manually screened the documents retrieved from the different sources based on the inclusion criteria. The titles and executive summaries of documents (where applicable) were screened to identify policy documents that cut across the entire health sector or were immunization-specific and created since the establishment of GAVI in 2000 up until 2023. In addition, a search of terms including vulnerability, immunization, and vaccination within the documents was conducted. Documents that were for other health sector programmes and/or created before 2000 were excluded. The list of documents identified as eligible for review was then shared with the other reviewers (PC, CJ, DM and DK). The team met to discuss the appropriateness of the documents identified as eligible, and a consensus was reached on documents to be included for final review.

### V. Data collection process and synthesis

Data were extracted from the included documents using a data extraction tool ^16^ prepared as a template in Excel. Each row of the tool contained a document, while the individual columns captured the type of information to extract from the documents. Extracted data included general information about the documents, namely, the full title of the document, author(s), date of publication, intended users/audience, actors (persons, groups or organizations involved in and influencing the formulation and implementation of the document) and a summary of document content. In addition, information related to how vulnerability was defined, any components of vulnerability and strategies suggested to address vulnerability were identified and extracted. The extracted data were then organized thematically to address the review objectives; how vulnerability issues are conceptualized/defined, aspects considered in the documents and strategies proposed to address vulnerability issues.

## Results

### 1. Study selection

**Figure 1.**
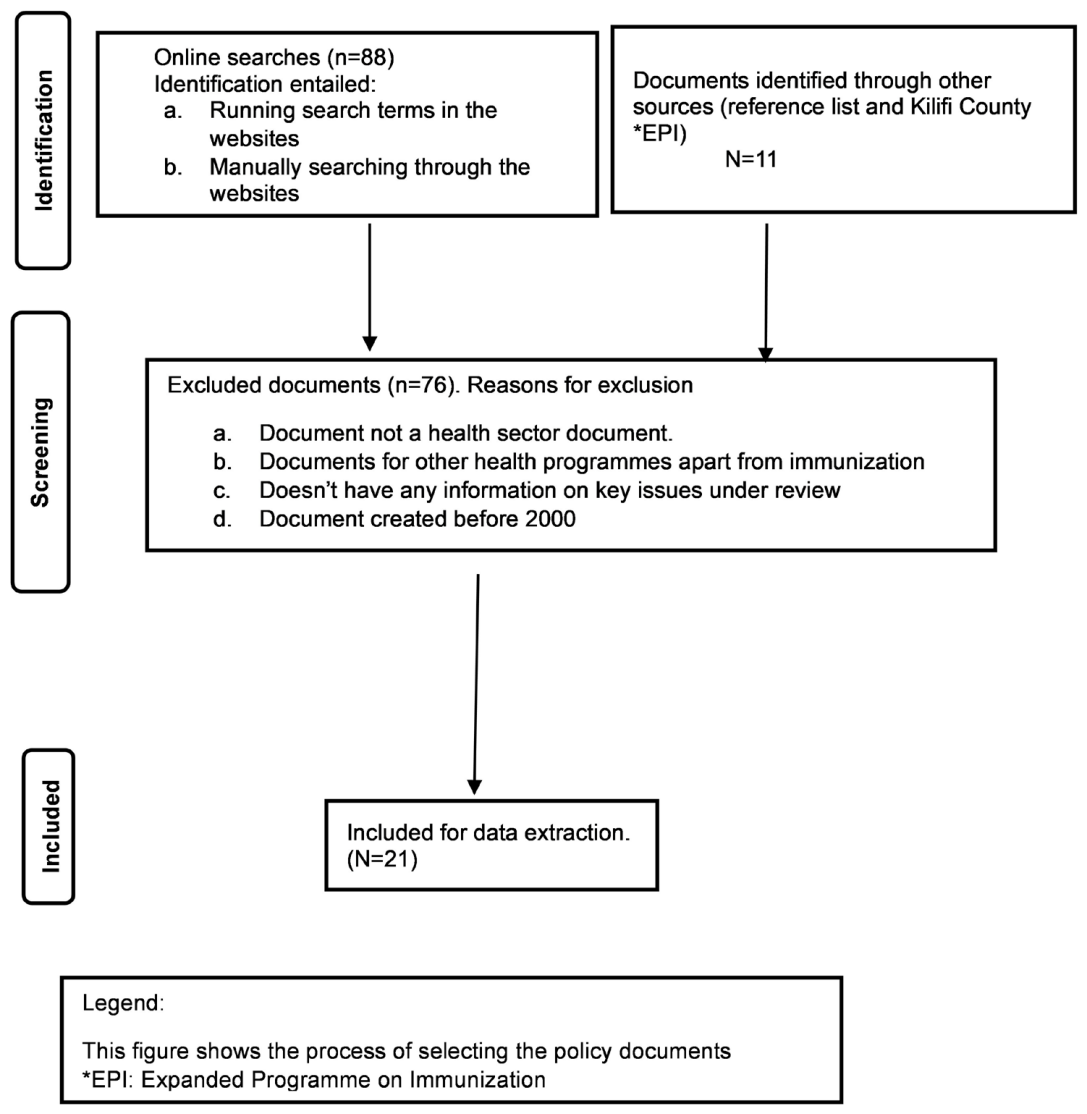
Flow of the document selection process. This figure shows the systematic process we followed to select the policy documents for review. *EPI: Expanded Programme on Immunization.

A total of 88 documents were retrieved through online searches, and an additional 11 from other sources, such as reference lists and stakeholders from the Kilifi County Department of Health. Of the 99 documents retrieved, 78 were excluded because they were either not health sector documents, for other health programmes apart from immunization, or the document didn’t have any information on key issues under review. Twenty-one documents were included for final review and data extraction. Figure 1 summarizes the process of document selection.

### 2. Categories of documents reviewed

A total of 21 policy documents were reviewed. Of these, 15 were categorised as health sector documents, 4 were immunization-specific documents, and two were legislative documents as summarized in Table 1.

**Table 1.** Policy documents reviewed. This table shows the list of policy documents included for review.

Category of document	Documents
Health sector documents	1. Kenya national eHealth policy 2016–2030 ^17^ 2. Kilifi county department of health and sanitation: Consolidated County annual workplan 2023/2024 ^18^ 3. Kenya health financing strategy 2020–2030 ^19^ 4. Kenya health information system policy ^20^ 5. Kenya health policy 2014–2030 ^21^ 6. Kenya health sector strategic plan 2018–2023 ^22^ 7. Kenya community health strategy 2020–2025 ^23^ 8. Kenya primary healthcare strategic framework 2019–2024 ^24^ 9. Kilifi county integrated annual workplan (AWP) financial year 2022/23 ^25^ 10. Strategy for community health 2014–2019 ^26^ 11. Sessional paper No.4 of 2012 on national pharmaceutical policy ^27^ 12. The Kenya health public private collaboration strategy 2020 ^28^ 13. The national health sector strategic plan of Kenya -NHSSP II 2005–2010 ^29^ 14. Transforming health systems for universal care (THS-UC) ^30^ 15. Kenya universal health coverage policy 2020–2030 ^31^
Immunization policy documents	1. National policy guidelines on immunization 2013 ^32^ 2. Division of vaccines and immunization (DVI) multiyear plan 2011–2015 ^33^ 3. Comprehensive multiyear plan for immunization July 2015–June 2019 ^34^ 4. Kenya national immunization policy guidelines 2023 ^35^
Legal/legislative documents	2. The constitution of Kenya 2010 ^36^ 3. The health ACT No. 21 of 2017 ^37^

### 3. Content of documents reviewed

**Figure 2.**
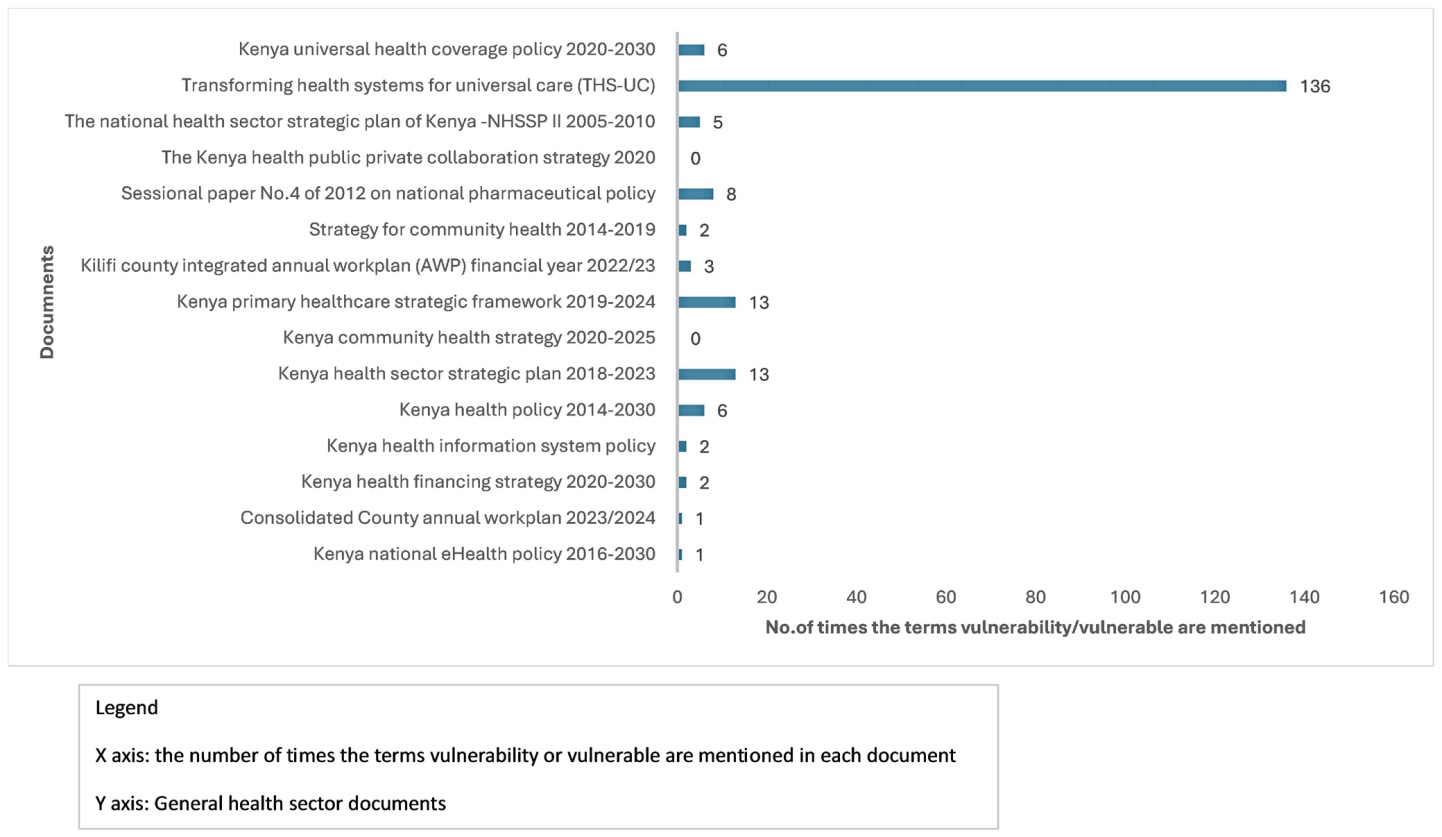
Number of times the terms vulnerability/vulnerable appear in reviewed general health sector documents. This table shows how many times the term vulnerability or vulnerable was mentioned in the general health sector policy documents reviewed.

***a. Content of general health sector documents.*** Of the 15 health sector documents reviewed, 13 explicitly mentioned the term/phrase vulnerable group/persons and/or vulnerability. In the other two documents, economic or social characteristics of groups of people, such as ‘the poor’, were used, but the term “vulnerable” and/or “vulnerability” were not mentioned. The documents in which the terms vulnerability, vulnerable groups and vulnerable persons were mentioned in the objectives, policy directions, targets and implementation plans included the *Transforming health systems for universal care: vulnerable and marginalized groups; the Kenya primary healthcare strategic framework 2019–2024* and the *Kenya health sector strategic plan 2018–2023 as shown in* Figure 2. A total of 136 mentions of the terms “vulnerable” and/or “vulnerability” were made in the *Transforming health systems for universal care: vulnerable and marginalized groups* document, 13 mentions in the *Kenya health sector strategic plan 2018–2023* and *Kenya primary healthcare strategic framework 2019–2024*.

In most of these health sector documents, while the terms were widely used, limited information was provided about issues facing the vulnerable groups or what contributed to their vulnerability. For instance, in the section on equitable access to health care, the *Kenya national eHealth policy 2016–2030*, states that eHealth will provide the health system with the ability to reach vulnerable population groups in low-resource settings. However, the document lacks details on who these vulnerable groups are. Similarly, in the *Kenya health financing strategy 2020–2030*, vulnerability is mentioned in the forward as well as the goal and objectives sections to indicate that there will be effective safety nets for the poor and the vulnerable, but there is no further discussion or specification of who is included in the ‘vulnerable’ or the safety nets to be put in place or how they will be realized. The T *ransforming health systems for vulnerable and marginalised groups plans 2020–2021* was the only document whose content exclusively focused on the vulnerable. The vulnerable groups discussed in the document were based on the World Bank’s operational policy 4.10 on indigenous people, also known as vulnerable and marginalised groups (VMGs). Examples of these groups were the Illchamus and Endorois of Baringo county, Ogiek of Mt. Elgon Forest, Malakote/Waliwana of Tana River County among others.

***b. Content of immunization specific documents.*** In the four immunization documents reviewed, there were also no detailed discussions about vulnerable groups. One of these documents was the most recent national immunization policy guideline ( *Kenya national immunization policy guidelines 2023*) and while there was mention of vulnerable groups, very little information was provided on the challenges that such groups might experience related to immunization services. Surprisingly, the terms vulnerable and vulnerability were not used in the comprehensive multiyear plans for immunization (CMYPs) 2011–2015 & July 2015–June 2019; the phrase used was “hard to reach populations”. In both documents, challenges that influence access and uptake of immunization were generally discussed. For example, in both the CMYPs, the following were highlighted as the general challenges to vaccine access and uptake: inadequate financing and human resources, inaccessibility of immunization services, missed opportunities, lack of technical capacity and communication challenges to create demand for immunization services.

***c. Content of legislative policy documents.*** The legislative documents reviewed in this study were the *Kenyan Constitution 2010* and the *Health Act No. 21 of 2017*. In the constitution, the term vulnerability was captured in Article 20 on the application of the Bill of Rights and Article 21, which focuses on the implementation of rights and fundamental freedoms. Article 20(5b) states that *“in allocating resources, the State shall give priority to ensuring the widest possible enjoyment of the right or fundamental freedom having regard to prevailing circumstances, including the vulnerability of particular groups or individuals.”* Further under Article 21(3) on the implementation of rights and fundamental freedoms, women, older members of society, persons with disabilities, children, youth, members of minority or marginalised communities, and members of certain ethnic, religious or cultural communities were listed as vulnerable groups. The Health Act No. 21 of 2017 reiterates the provisions of the vulnerable groups as enshrined in Article 21 of the constitution, which states that *“all State organs and all public officers have the duty to address the needs of vulnerable groups within society...”*

### 2. Framing/Conceptualization of vulnerability in the reviewed documents

In the *Kenya primary healthcare strategic framework 2019–2024* vulnerability was defined as *“the degree to which a population, individual or organization is unable to anticipate, cope with, resist and recover from the impacts of disasters”* while vulnerable populations were defined as *“a group of people such as children, pregnant women, elderly people, malnourished people, and people who are ill or immunocompromised, socioeconomically disadvantaged who are particularly vulnerable when a disaster strikes, and take a relatively high share of the disease burden associated with emergencies”.* A common trend across the other documents was the mention of groups considered vulnerable, as summarized in Table 2 (extended data), implying that vulnerability was associated with certain groups of people. Whereas none of the documents defined different types of vulnerability, we developed four typologies of vulnerability from the groups listed in the documents, namely vulnerability as a socio-economic condition, vulnerability as a biological condition, vulnerability as a health condition and vulnerability as a physical location.

***a. Vulnerability as a socio-economic condition.*** Across all the documents reviewed, vulnerability seemed to emanate from people’s socio-economic positions/conditions. This was depicted by a range of socio-economic factors implied by the vulnerable groups listed across the policy documents. The factors included ethnicity, education, occupation, social class and income. As such, the most common vulnerable groups mentioned in the documents were the poor, sex workers, the less educated, refugees, nomadic populations, and those living in informal settlements. Other broad groups namely marginalized communities and minorities were also used, but specific groups under these were not provided.

***b. Vulnerability as a biological condition.*** A second typology of vulnerability implied in the reviewed documents relates to physical and physiological conditions. The main factors associated with this type of vulnerability included age and physical disability. Some of the vulnerable groups categorized under this included the elderly, children, adolescents, women and persons living with disability.

***c. Vulnerability as a health condition.*** In the documents, vulnerability was also implied to emanate from health conditions. As such, individuals suffering from certain specific diseases were considered vulnerable. Categories of vulnerable individuals under this typology include chronic health conditions like HIV, malnourished individuals, and those who abuse drugs.

***d. Vulnerability as a physical location/vulnerability of places.*** Lastly, vulnerability was associated with certain geographical locations. Thus, communities and/or population groups residing in those locations were considered vulnerable. Examples of these included far-to-reach/hard-to-reach areas, remote/rural areas, urban informal settlements and insecurity affected settings. However, unlike the two other types of vulnerability, this third type was only prominent in the immunization-specific documents compared to the general health sector documents, where only five documents identified vulnerable groups according to their geographical location. None of the legislative documents identified vulnerable groups under this category.

### 1. Measures/strategies to address vulnerability issues

Various measures were proposed in the reviewed documents to address the needs of the groups that had been categorized as vulnerable. We grouped the key measures into the following categories:

***a. Rights-based approach.*** A rights-based approach to provision of health services was proposed to ensure that health related rights of the vulnerable are respected and protected. This was proposed in the following documents: the Health Act No.21 of 2017, Kenya Health Policy, the National Health Sector Strategic Plan 2005–2010 and UHC policy 2020–2030. The Health Act No.21 of 2017 stipulates that the rights of vulnerable groups should be protected, respected and promoted as per Article 21 of the Kenyan constitution, ensuring that their health-related rights and interests are realized. In the Kenya Health Policy 2014–2030, one of the obligations for the policy is a progressive realization of rights to health, as illustrated in the clause below.

*“The national and county governments will put in place measures to progressively realise the right to health as outlined in Article 21 of the Constitution. The sector will employ a human rights-based approach in healthcare delivery and will integrate human rights norms and principles in the design, implementation, monitoring, and evaluation of health interventions and programmes. This includes human dignity; attention to the needs and rights of all, with special emphasis on children, persons with disabilities, youth, minorities and marginalised groups, and older members of the society (Constitution of Kenya 2010 Article 53–57); and ensuring that health services are made accessible to all.”* Kenya health policy 2014–2030; pg 30.

***b. Research to support evidence-based policy for the vulnerable and their health needs.*** A second measure proposed was conducting research focusing on the needs of the vulnerable. The Health Act No.21 of 2017 states that due regard will be paid to the health needs of the vulnerable when identifying priorities for health research. In the Kenya health policy, one of the policy orientations on research and development specifically states that *“MOH will prioritise research in order to support evidence-based policy and intervention formulation, identifying gaps and critical factors for special needs for vulnerable groups especially the women, children and the elderly*” Kenya health policy 2014–2030; pg 51 *.* Closely related to research was the proposal to conduct risk mapping and vulnerability assessments to manage emergency disasters that may negatively affect vulnerable groups (Kenya Health Sector Strategic Plan and Transforming Health System: Achieving Universal Health Coverage by 2022).

***c. Removing financial barriers to health access by vulnerable groups.*** Another measure captured in some of the documents is the reduction of financial barriers that hinder vulnerable populations/groups from accessing health services. In the Sessional Paper No. 4 of 2012 on National Pharmaceutical Policy, this could be achieved by progressively eliminating user fee payments at the point of health service use for vulnerable groups, health insurance and subsidies. Further strategies were proposed in the National Health Sector Strategic Plan 2005–2010 and these included making investments that benefit the poor, improving resource allocation and financial targeting to underserved and poor areas as well as a gender focus¸ increasing funding as part of pro-poor agenda, define datasets to measure regional disparities, developing common financial tools and renting out underutilized facilities to private providers to cushion the vulnerable from high cost of health care.

***d. Strengthening access to primary health services.*** In the Kenya primary healthcare strategic framework, one of the key objectives was to strengthen access to primary health services for the vulnerable groups. This was to be attained by developing a health promotion framework as well as guidelines, policies and standards for vulnerable population; establishing community rehabilitation, palliative, rescue centres and home-based care and enhancing their linkages with facility-based rehabilitation, palliative care centres, the community and other relevant sectors.

***e. Conducting outreach activities.*** All the immunization policy documents reviewed identified conducting integrated outreaches as the main measure proposed to reach the vulnerable groups. For instance, in the national policy guidelines on immunization 2013 and 2023, the only explicit strategy to increase access for groups considered as vulnerable, such as zero-dose and under vaccinated children is to conduct targeted immunization outreach activities integrated with other maternal and child survival services in areas known to have such groups.

## Discussion

This review examines how vulnerability and the vulnerable have been conceptualised in health policy documents in Kenya, focusing on immunization-specific policy documents. Most of the policy documents reviewed outline vulnerable groups. The identified groups imply four types of vulnerabilities: vulnerability arising from socioeconomic, biological, and health condition(s)/situation(s); and vulnerability associated with certain physical locations. These four typologies suggest a sensitivity to vulnerability issues and align with definitions used in previous vulnerability studies within the context of health. Grabovschi *et al.* 2013, in a scoping review mapping the concept of vulnerability related to health care disparities, operationalised vulnerability as an increased susceptibility to health and health care disparities due to both individual and environmental factors in the immediate physical environment and the broader socioeconomic environment. However, as outlined in the documents, the homogeneous categorisation of individuals as belonging to certain vulnerable groups has been heavily criticised. Labelling certain groups as vulnerable without being explicit about how such categorisation has been reached has been argued to downplay their agency, leading to further stigmatization and exclusion. Additionally, such groupings imply automatic vulnerability for anyone falling into the identified categories and assume that vulnerability is a permanent condition.

In recent years, attempts have been made to address concerns regarding the categorization of individuals as vulnerable and to broaden the conceptualization of vulnerability within the policy context. Kuran and colleagues ^38^ recommend employing an intersectionality lens to interrogate the concept, given the intertwining and intersecting nature of the factors that contribute to vulnerability. For instance, vulnerability studies in the health context have demonstrated that individuals and households typically experience a multiplicity of vulnerabilities ^38– 41^. Therefore, adopting an intersectionality lens necessitates recognizing the multiple factors that can influence the lived experiences of individuals and groups. Prioritizing a single factor or a set of factors overlooks the multiplicity and intersecting influences that shape vulnerability ^17, 18, 42 43^. In the policy documents reviewed for this paper, the vulnerabilities of specific groups were defined in relation to individual factors without acknowledging the potentially overlapping and interacting nature of these factors. Groups such as the poor, women, and people living in informal settlements were cited as separate entities in the documents, failing to recognize the overlapping factors at an individual level. Adopting an intersectionality lens would facilitate the identification and description of the various forms of vulnerabilities responsible for inequities in access to healthcare services, such as immunization. In the future, policy development should strive to move beyond simplistic categorizations of vulnerable individuals by single-factor groups and incorporate consideration of the multiplicity and intersecting nature of different factors that contribute to vulnerability in particular situations.

The policy documents reviewed referenced the need to consider vulnerable groups, as such measures to address the needs of these groups were spelt out broadly. One of the measures was a rights-based approach to the provision of health care services. This can be seen as a step in the right direction as such an approach recognizes that health is a fundamental human right and obligates healthcare providers to ensure that this right is respected, protected and fulfilled for everyone. However, guiding how this would be attained is important. For immunization-specific strategies, conducting integrated outreach activities was the main measure recommended for reaching vulnerable groups. Whereas this strategy would help bring immunization services closer to those experiencing physical accessibility challenges to health facilities, it may not address other forms of vulnerabilities highlighted in primary studies, including household, caregiver and health system level vulnerabilities. For instance, in Kenya, previous studies have shown that children’s vulnerability to vaccine-related disparities is not only influenced by geographical factors, but also household and caregivers’ characteristics ^8, 10, 38– 40, 44, 45^. Therefore, a more comprehensive and flexible approach would be suitable to addressing the different forms of vaccine-related vulnerabilities.

Considering the findings of this review and our proposal for an intersectional lens, policy makers in the health sector can prioritize providing guidance and guidelines on how to assess and identify health related vulnerabilities and possible measures to address them. First, this can be in the form of tools and training manuals aimed at capacity building of healthcare workers to identify vulnerabilities in their contexts and develop more appropriate contextual measures to address the same. Bourgois *et al.* 2017 ^41^ proposed a structural vulnerability assessment tool for quick screening of patients to enable health care practitioners in the American context to prioritize comprehensive treatment plans that interface with resources outside the clinic. The tool has screening questions under eight domains namely, financial security, place of residence, risk environments, food access, social network, legal status, education and discrimination. Whereas the tool was more oriented towards a high-income country, such can be modified or act as a starting point to developing tools more appropriate to the Kenyan context. Use of such tools can help promote an understanding among health care workers on how various factors can undermine the capacities of patients’ access and use of health care services.

Second, given that vulnerability isn’t static but changes with time ^42^, health-related data as well as local contextual knowledge should be regularly used in planning for health services. This can help to update the vulnerability focus by regularly reviewing those who should be prioritized, when and with which activities. For instance, Utazi *et al.* 2023, used data on risk factors for zero dose and under-immunization, as well as vaccination coverage, from demographic and health surveys conducted in the Democratic Republic of Congo, Ethiopia, India, Pakistan, and Uganda as well as a national immunization coverage survey conducted in Nigeria to develop a vulnerability index for identifying zero-dose children. In the broader study within which this review is embedded, different Kenyan and Ugandan health data sets were also used to develop a vulnerability index to assess the level of a community's vulnerability to vaccine impact. Such vulnerability indexes can be updated periodically as new data is collected to inform who should be prioritized. In addition, primary qualitative studies can also be crucial in collecting data relevant to specific context(s) hence allowing for more targeted approaches tailored to address specific vulnerabilities.

### Limitations

Whereas this document review was not a systematic review of published literature, it highlights the current state of vulnerability issues in the context of healthcare policy in Kenya; information that can be useful in improving the current policies to be more comprehensive. Although every effort was made to secure all relevant documents for the review, we recognise that we might not have included all the relevant policy documents because the majority of the included documents were those publicly available online. In addition, we were only able to receive few documents from stakeholders.

## Conclusions

In this review, we have shown that vulnerability in the healthcare policy context in Kenya is primarily associated with socio-economic, behavioural and health conditions, as well as specific physical locations. However, the intersecting nature of these vulnerabilities has not been recognised. In light of these findings, we recommend employing an intersectionality lens to guide the development of more comprehensive policies and strategies. These may include capacity building for health workers on using data and vulnerability assessment tools that key stakeholders within the health and immunization landscape can use to identify and better target immunization services for populations with limited access and uptake of such services.

## Data Availability

Harvard Dataverse: To what extent are vulnerability issues included and addressed in health and immunization policy documents in Kenya? A systematic review of documents ^43^ https://doi.org/10.7910/DVN/BC633W. Extended data file 1: PRISMA checklist Extended data file 2: Extracted data Extended data file 3: Summary of vulnerable groups listed in the documents Data are available under the terms of the Creative Commons Attribution 4.0 International License (CC BY 4.0). We have completed a PRISMA checklist and uploaded it on Harvard Dataverse https://doi.org/10.7910/DVN/BC633W ^43^ Data are available under the terms of the Creative Commons Attribution 4.0 International License (CC BY 4.0).
