# Supplementary material for: To what extent are vulnerability issues included and addressed in Kenya's health and immunization policy documents? A systematic review of documents
Source: NIHR Open Res. 2025 Aug 1;5:63. [Version 1] doi: 10.3310/nihropenres.14010.1 (PMC12648031; doi:10.3310/nihropenres.14010.1)
Supplement: Peer review history

# Reviewer report: Reviewer response for version 1

**DOI:** 10.3310/nihropenres.15233.r38191

**Published:** 2025-11-25

**Authors:** Rita Barradas Barata

**Affiliations:** 1 Faculdade de Ciências Médicas da Santa Casa de São Paulo, São Paulo, Brazil

The article “To what extent are vulnerability issues included and addressed in Kenya’s health and immunization policy documents? A systematic review of documents” presents a review of health policy documents focusing on a particular issue: vulnerability.

 In general, the article is very well written and correctly conducts the methodological steps related to document eligibility criteria, source identification, search strategies, document selection, and analysis of the contents of interest.

 Two theoretical and one methodological aspect deserve highlighting. From a methodological point of view, the choice of the PRISMA tool does not seem entirely appropriate given the characteristics of the documents that constituted the object of analysis. The tool is better suited to the synthesis of empirical research than to document analysis. In any case, the choice did not result in concrete problems for the authors, although other approaches to content analysis in documental research might be more appropriate.

 From a theoretical standpoint, I would like to highlight two issues related to the vulnerability construct. Firstly, the authors choose to work with a concept of vulnerability developed for the area of ​​environmental disasters instead of resorting to the concept developed in Public Health, which would be more appropriate for the object of this research.

 The concept of vulnerability in Public Health, was subjected to a expanded and theoretically consistent formulation during the AIDS epidemic, using several dimensions to comprehensively understand the epidemic process, the epidemiological situation and the health practices required to meet that challenge. This concept, proposed by Jonathan Mann and Tarantola, draws attention to a range of individual, collective and contextual factors. The concept of vulnerability encompasses three dimensions of the health-disease production process: individual characteristics, social process and aspects of health police, programs and services, which together affect both the epidemiological profile and the access and use of the health services. Throughout the text there are moments of confusion between susceptibility and vulnerability, probably due to a lack of clarity stemming from the choice regarding the concept of vulnerability.

 Secondly, the authors also lack a clear position regarding immunization programs specifically. Immunization programs should always be motivated by the concept of universality, that is, aiming for universal population coverage so that the benefits of herd immunity can be achieved through immunizations performed on individuals. To achieve this goal, it will be necessary to combine mass approaches (everyone should be immunized) with specific strategies aimed at reaching groups that, for various reasons, cannot directly benefit from mass proposals, whether due to individual, social, or programmatic vulnerability. By not correctly identifying the different dimensions of vulnerability and mixing them with inequalities and heterogeneity in coverage, there is no explicit vision of the need to reach everyone, universally, to obtain the expected results, that is, the control of vaccine-preventable diseases.

 A clearer understanding of the problems would also allow for a clearer organization of the different intervention strategies. Starting from the need for universal coverage, barriers related to access, provision, and organization of services could be considered both at the community level and for particularly vulnerable subgroups.

Are the rationale for, and objectives of, the Systematic Review clearly stated?

Yes

Is the statistical analysis and its interpretation appropriate?

Not applicable

Are sufficient details of the methods and analysis provided to allow replication by others?

Yes

Are the conclusions drawn adequately supported by the results presented in the review?

Yes

Reviewer Expertise:

Collective Health

I confirm that I have read this submission and believe that I have an appropriate level of expertise to confirm that it is of an acceptable scientific standard.

# Reviewer report: Reviewer response for version 1

**DOI:** 10.3310/nihropenres.15233.r36929

**Published:** 2025-10-07

**Authors:** Lorena G. Barberia

**Affiliations:** 1 University of São Paulo, São Paulo, Brazil

This is an important study which seeks to carefully examine how the Kenyan government has defined "vulnerability" in the context of child immunization in policy documents. The study helps to map gaps in how vulnerability is understood by showing that the definition of vulnerability varies across documents and emphasizes specific conditions.  The review presents results showing that individuals in certain categories are classified as vulnerable, and these classifications can vary or be too restricted considering that there are contexts in which vulnerability changes over time even within the same disease threat. This type of review is important as it can be used in future work to evaluate if government programs are targeting vulnerabilities.

Are the rationale for, and objectives of, the Systematic Review clearly stated?

Yes

Is the statistical analysis and its interpretation appropriate?

Not applicable

Are sufficient details of the methods and analysis provided to allow replication by others?

Yes

Are the conclusions drawn adequately supported by the results presented in the review?

Yes

Reviewer Expertise:

Vaccine Uptake; Vaccine hesitancy: Vaccine coverage; Policy Evaluation

I confirm that I have read this submission and believe that I have an appropriate level of expertise to confirm that it is of an acceptable scientific standard.
